# The value of ultrasound-defined tenosynovitis and synovitis in the prediction of persistent arthritis

**DOI:** 10.1093/rheumatology/keac199

**Published:** 2022-04-12

**Authors:** Ilfita Sahbudin, Ruchir Singh, Paola De Pablo, Elizabeth Rankin, Benjamin Rhodes, Elizabeth Justice, Emma Derrett-Smith, Nicole Amft, Nehal Narayan, Catherine McGrath, Sangeetha Baskar, Jeanette Trickey, Mark Maybury, Karim Raza, Andrew Filer

**Affiliations:** Rheumatology Research Group, Institute of Inflammation and Ageing; Research into Inflammatory Arthritis Centre, MRC Versus Arthritis Centre for Musculoskeletal Ageing Research, University of Birmingham; NIHR Birmingham Biomedical Research Centre, University Hospitals Birmingham NHS Foundation Trust; Rheumatology Research Group, Institute of Inflammation and Ageing; Research into Inflammatory Arthritis Centre, MRC Versus Arthritis Centre for Musculoskeletal Ageing Research, University of Birmingham; Department of Rheumatology, Sandwell and West Birmingham Hospitals NHS Trust; Rheumatology Research Group, Institute of Inflammation and Ageing; Research into Inflammatory Arthritis Centre, MRC Versus Arthritis Centre for Musculoskeletal Ageing Research, University of Birmingham; Department of Rheumatology, Sandwell and West Birmingham Hospitals NHS Trust; Rheumatology Department, University Hospitals Birmingham NHS Foundation Trust, Birmingham, UK; Rheumatology Department, University Hospitals Birmingham NHS Foundation Trust, Birmingham, UK; Rheumatology Department, University Hospitals Birmingham NHS Foundation Trust, Birmingham, UK; Rheumatology Department, University Hospitals Birmingham NHS Foundation Trust, Birmingham, UK; Rheumatology Department, University Hospitals Birmingham NHS Foundation Trust, Birmingham, UK; Rheumatology Department, University Hospitals Birmingham NHS Foundation Trust, Birmingham, UK; Department of Rheumatology, Sandwell and West Birmingham Hospitals NHS Trust; Department of Rheumatology, Sandwell and West Birmingham Hospitals NHS Trust; Rheumatology Research Group, Institute of Inflammation and Ageing; Research into Inflammatory Arthritis Centre, MRC Versus Arthritis Centre for Musculoskeletal Ageing Research, University of Birmingham; NIHR Birmingham Biomedical Research Centre, University Hospitals Birmingham NHS Foundation Trust; Rheumatology Research Group, Institute of Inflammation and Ageing; Research into Inflammatory Arthritis Centre, MRC Versus Arthritis Centre for Musculoskeletal Ageing Research, University of Birmingham; NIHR Birmingham Biomedical Research Centre, University Hospitals Birmingham NHS Foundation Trust; Rheumatology Research Group, Institute of Inflammation and Ageing; Research into Inflammatory Arthritis Centre, MRC Versus Arthritis Centre for Musculoskeletal Ageing Research, University of Birmingham; Department of Rheumatology, Sandwell and West Birmingham Hospitals NHS Trust; Rheumatology Research Group, Institute of Inflammation and Ageing; Research into Inflammatory Arthritis Centre, MRC Versus Arthritis Centre for Musculoskeletal Ageing Research, University of Birmingham; NIHR Birmingham Biomedical Research Centre, University Hospitals Birmingham NHS Foundation Trust

**Keywords:** early arthritis, ultrasound, prediction, persistent arthritis

## Abstract

**Objectives:**

The value of US-defined tenosynovitis in predicting the persistence of inflammatory arthritis is not well described. In particular, the predictive utility of US-defined tenosynovitis of larger tendons is yet to be reported. We assessed the value of US-defined tenosynovitis alongside US-defined synovitis and clinical and serological variables in predicting persistent arthritis in an inception cohort of DMARD-naïve patients with early arthritis.

**Methods:**

One hundred and fifty DMARD-naïve patients with clinically apparent synovitis of one or more joints and a symptom duration of ≤3 months underwent baseline clinical, laboratory and US (of 19 bilateral joints and 16 bilateral tendon compartments) assessments. Outcomes were classified as persistent or resolving arthritis after 18 months’ follow-up. The predictive value of US-defined tenosynovitis for persistent arthritis was compared with those of US-defined synovitis, and clinical and serological variables.

**Results:**

At 18 months, 99 patients (66%) had developed persistent arthritis and 51 patients (34%) had resolving disease. Multivariate logistic regression analysis showed that US-detected digit flexor tenosynovitis [odds ratio (OR): 6.6, 95% CI: 2.0 , 22.1, *P* = 0.002] provided independent predictive data for persistence over and above the presence of US-detected joint synovitis and RF antibodies. In the RF/ACPA-negative subcohort, US-defined digit flexor tenosynovitis remained a significant predictive variable (OR: 4.7, 95% CI: 1.4, 15.8, *P* = 0.012), even after adjusting for US-defined joint synovitis.

**Conclusion:**

US-defined tenosynovitis provided independent predictive data for the development of persistent arthritis. The predictive role of US-defined digit flexor tenosynovitis should be further assessed; investigators should consider including this tendon site as a candidate variable when designing imaging-based predictive algorithms for persistent inflammatory arthritis development.

Rheumatology key messages

- Ultrasound-defined digit flexor tenosynovitis is an independent predictor of persistent arthritis in early arthritis patients.
- Prediction of persistence by ultrasound-defined digit flexor tenosynovitis is independent of synovitis and clinical variables.
- Clinicians should consider scanning digit flexor tendons alongside joints in patients with early arthritis.

## Introduction

There is a window of opportunity in early arthritis during which immunosuppressant intervention can change the trajectory of the disease in inflammatory arthritis [1–5]. Therefore, there is a need to develop an enhanced set of validated tools that clinicians can use to identify patients at risk of developing persistent arthritis. This is vital so that DMARDs can be targeted to the correct patients early in their disease course [6, 7].

Current predictive algorithms focus on clinical features (e.g. patterns of joint involvement, symptom duration) and serological variables (e.g. inflammatory markers, autoantibodies) as predictors of persistent inflammatory arthritis [6, 8]. More recently, studies have assessed the utility of US imaging features in prediction models for persistent arthritis, given the ability of US to identify joint inflammation that is not clinically apparent [9]. However, US variables included in such predictive studies were predominantly joint synovitis variables [10–13].

At present, the role of tenosynovitis (TS) in the prediction of persistent inflammatory arthritis has not been described. In particular, the predictive utility of US-defined TS related to the larger joints has yet to be reported. US is a reliable and easily accessible tool for detecting tendon inflammation in patients with inflammatory arthritis [14]. In addition, US is an increasingly available imaging modality in rheumatology departments, and access to training is more widespread [15, 16].

We previously reported that US-defined TS improved the prediction of RA independently of US-defined synovitis, and clinical and serological variables in patients with early arthritis [17].

In the current work, we sought to describe the prevalence of US-detected joint and tendon inflammation involving both small and large joints in a cohort of patients presenting with inflammatory arthritis and a symptom duration of 3 months or less. Second, we investigated whether US synovial and tenosynovial variables independently predict persistent arthritis development, above and beyond clinical and serological predictors.

## Methods

### Patients and clinical assessment

Patients were recruited to the Birmingham Early Arthritis Cohort (BEACON) from early arthritis clinics at Sandwell and West Birmingham NHS Trust and University Hospitals Birmingham NHS Foundation Trust, UK. All patients were referred by their GP to these two secondary care centres. Consecutive DMARD-naïve patients with clinically detected synovitis of at least one joint and inflammatory joint symptoms (pain and/or stiffness and/or swelling) of 3 months’ duration or less were included. Patients with joint symptoms attributed solely to degenerative joint disease were excluded. All consecutive patients who consented to this study were included in the analysis except for those who declined to continue follow-up before final diagnostic outcome data were available. The following data were recorded at baseline: 68 tender and 66 swollen clinical counts, age, sex, symptom duration, early morning stiffness duration, medication, ESR, CRP, RF and ACPA status.

Patients were classified as having persistent arthritis or resolving arthritis at the 18-months follow-up. Patients were classified as having resolving disease if they had no clinical evidence of synovial swelling, were not taking DMARDs and had not received DMARD or steroid treatment for joint disease in the previous 3 months. Patients with persistent arthritis were classified based on established classification criteria: 2010 ACR/EULAR classification criteria for RA [18] or 1987 ACR classification criteria for RA [19], Classification Criteria for Psoriatic Arthritis (CASPAR) [20], SLICC classification criteria for SLE [21], 2015 ACR/EULAR Gout Classification Criteria [22], Assessment of SpondyloArthritis International Society (ASAS) classification criteria for peripheral SpA and SpA in general [23], and diagnostic criteria for reactive arthritis [24]. Palindromic arthritis was defined as history or physical examination findings consistent with synovial swelling that returned to normal between episodes. Patients with septic arthritis, pseudo-gout and sarcoidosis were classified based on clinical diagnosis. This study was approved by the West Midlands—Black Country Research Ethics Committee (12/WM/0258), and written informed consent was obtained from all participants.

In this observational study, patients who required disease-modifying therapy were treated according to standard-of-care practice. Conventional synthetic DMARDs were first-line therapy, consistent with National Institute for Health and Care Excellence (NICE) guidelines.

### Sonographic assessment

Within 24 h of clinical assessment, an experienced sonographer (A.F. or I.S.) performed a blinded US assessment in a temperature-controlled radiology suite. Systematic multi-planar grayscale (GS) and power Doppler US examinations were performed based upon standard EULAR reference scans [25] using a Siemens Acuson Antares scanner (Siemens, Bracknell, UK) with multifrequency (5–13 MHz) linear array transducers, GE S8 (Milwaukee USA) or E9 (Milwaukee USA) with multifrequency (6–15 MHz) linear array transducers. The machines were centrally calibrated for GS and power Doppler settings. The joint and tendon recesses scanned are listed in Supplementary Tables S1 and S2, available at *Rheumatology* online, respectively.

A total of 150 patients underwent US assessment of bilateral MCP 1–5, PIP 1–5, wrists and MTP 2–5 synovial joints. Of these, 107 patients also had US assessment of bilateral elbow, shoulder, ankle and knee tenosynovial and synovial joints. In addition, 113 out of the 150 patients had bilateral digit flexor, wrist flexor and wrist extensor compartment tendons scanned, of whom 111 had the full six-compartment wrist extensor tendon set and two patients had extensor carpi ulnaris (ECU) tendon scans only.

For power Doppler examinations, the pulse repetition frequency (PRF) was adjusted to provide maximal sensitivity at the lowest possible value for each joint, resulting in PRFs of between 610 and 780. Examinations took between 40 and 60 min depending on disease extent and patient mobility.

US findings of GS synovial hypertrophy and power Doppler positivity were defined according to consensus definitions. GS and power Doppler positivity in the MCP, PIP and MTP joints were graded from 0 to 3 as per consensus definition [9, 26]. Synovitis in other joints was graded as 0, normal; 1, mild; 2, moderate; and 3, severe, as previously reported [27].

GS and power Doppler TS changes were defined and graded according to the OMERACT Ultrasound Task Force consensus definitions [14]. GS TS was defined as abnormal anechoic and/or hypoechoic (relative to tendon fibres) tendon sheath widening that was related to tenosynovial abnormal fluid and/or hypertrophy. Power Doppler TS was defined as the presence of peritendinous Doppler signal within the synovial sheath, seen in two perpendicular planes, excluding normal feeding vessels. For the analysis, all GS and power Doppler US variables were binarized into absent (grade = 0) or present (grade ≥ 1).

### Statistical analysis

All data analyses were performed using IBM SPSS Statistics for Windows (Version 26.0; IBM Corp., Armonk, NY, USA).

### Reliability analysis

Intraobserver reliability was evaluated by blindly rescoring representative images of 20 patients for joint US assessments, and analysed using κ statistics. Interobserver reliability was evaluated by blindly rescoring representative images of 20 patients by the two sonographers for joint US assessments, and analysed using κ statistics. A κ value of 0–0.2 was considered poor, 0.21–0.40 fair, 0.41–0.6 moderate, 0.61–0.8 good, and 0.81–1 excellent. The results of the reliability assessments are listed in Supplementary Tables S3–S5, available at *Rheumatology* online.

### Descriptive analysis

Baseline clinical variables were compared between groups (i.e. persistent arthritis or resolving arthritis at the 18-month follow-up) using Mann–Whitney or Fisher’s exact tests as appropriate. The proportion of patients with US-defined synovitis and TS was compared between the outcome groups using Fisher’s exact test. In descriptive analyses, a *P*-value of *P* ≤ 0.05 was considered statistically significant.

### Logistic regression and principal component analyses

The primary aim of this study was to identify the combination of US, and clinical and serological variables that were predictive of persistent inflammatory arthritis development. First, univariate logistic regression analysis was performed to identify individual baseline variables associated with persistent arthritis development. Second, principal component analysis (PCA) was used to assess the extent of clustering among US joint and tendon variables, and then clinical and serological variables.

The variable with the highest loading factor from each component was extracted and made available as an independent variable in a forward stepwise multivariate logistic regression analysis, with persistent arthritis outcome at 18 months entered as the dependent variable. All independent clinical and serological variables were classified into categories as listed in Supplementary Table S6, available at *Rheumatology* online, for persistent arthritis prediction.

## Results

### Demographic and disease characteristics

One hundred and fifty patients were included in this analysis. At 18 months, 99 (66%) developed persistent arthritis, and the remaining 51 patients (34%) had resolving disease. Patients with persistent arthritis were more likely to be older and reported longer symptom and early morning stiffness durations. More persistent arthritis patients had elevated levels of RF and ACPA antibodies, and they had higher tender and swollen joint counts at baseline. Seronegative persistent arthritis patients reported more prolonged symptom and early morning stiffness durations. Baseline characteristics by prognostic outcomes of all patients and seronegative patients are shown in Table 1 and Supplementary Table S7 (available at *Rheumatology* online), respectively.

**Table 1:** Baseline characteristics according to outcome group all patients (*n* = 150)

	Resolving inflammatory arthritis	Persistent inflammatory arthritis	*P*
***N***	51	99	
**Age, years**	45 (35–58)	57 (45–66)	0.008^b^
**Female, *n* (%)**	30 (58.8)	55 (55.6)	0.731^a^
**Symptom duration, weeks**	5 (4–8)	7 (5–9)	0.006^b^
**Early morning stiffness** ^c^ **, min**	30 (0–60)	90 (30–180)	<0.001^b^
**ACPA, *n* (%)**			
** Negative**	47 (92.2)	64 (64.6)	<0.001^a^
** Low positive**	0 0	3 (3.0)	
** High positive**	4 (7.8)	32 (32.3)	
**RF^d^, *n* (%)**			
** Negative**	46 (92.2)	58 (58.6)	<0.001^a^
** Low positive**	1 (2.0)	16 (16.2)	
** High positive**	3 (5.9)	25(25.3)	
**Mode of onset^e^, *n* (%)**			
** Acute**	36 (78.3)	60 (65.9)	0.168^a^
** Insidious**	10 (21.7)	31 (34.1)	
**NSAID use, *n* (%)**	30 (58.8)	65 (65.7)	0.475^a^
**ESR^c^, mm/h**	18 (5–33)	23 (10–43)	0.118^b^
**CRP^c^, mg/l**	8 (1–24)	15 (5–32)	0.110^b^
**Tender joint count of 68** ^f^	4 (1–7)	11 (3–19)	<0.001^b^
**Swollen joint count of 66**	2 (1–6)	6 (3–13)	<0.001^b^
**Tender joint count of 28**	2 (1–5)	7 (2–13)	<0.001^b^
**Swollen joint count of 28**	2 (1–4)	5 (2–11)	<0.001^b^
**DAS-28 CRP^f^**	3.45 (2.98–4.56)	4.75 (3.59–5.51)	<0.001^b^
**DAS-28 ESR** ^e^	3.84 (3.03–4.51)	4.91 (3.96–6.15)	<0.001^b^

All variables are shown as median (IQR) unless otherwise specified.

a Fisher’s exact test.

b Mann–Whitney test.

c *n* = 148.

d *n* = 149.

e *n* = 137.

f *n* = 147. DAS-28: DAS in 28 joints.

At the final time point, RA was the largest diagnostic subgroup among persistent arthritis patients, while unclassified arthritis was the largest subgroup among resolving arthritis patients. This was also the case for seronegative patients (Supplementary Table S8, available at *Rheumatology* online).

### Distribution and univariate logistic regression analysis of synovial US abnormalities

**Fig. 1:**
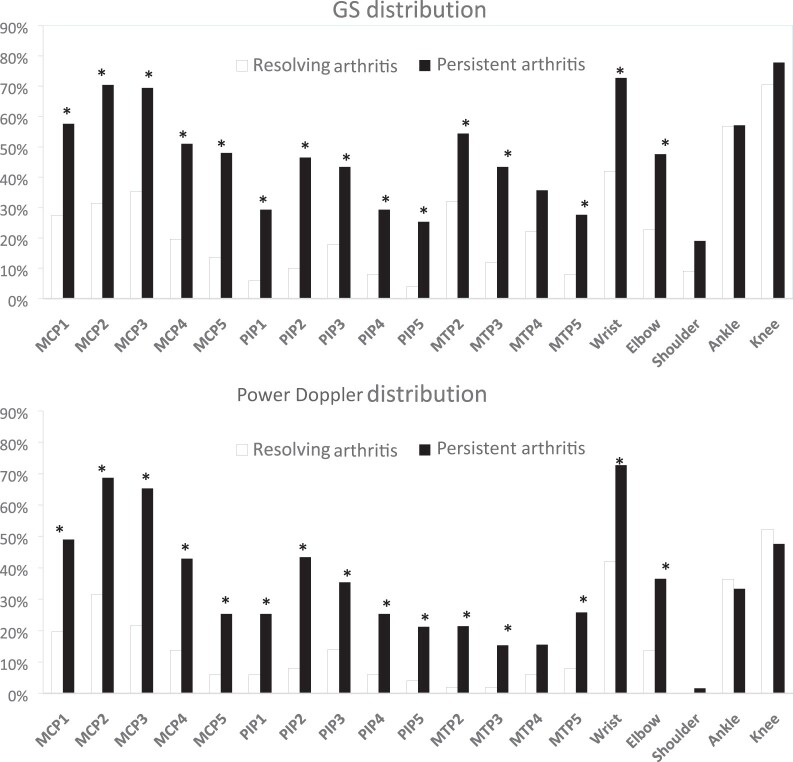
Distribution of joint US pathology (grayscale and power Doppler) for all patients (*n* = 150) Each bar represents the proportion of patients with US-defined synovitis involvement according to outcome groups. Data available for GS: *n* = 149; for MCP 2–5, PIP 1–5, MTP 2–3 and wrist; *n* = 148 for MTP 4–5; *n* = 107 for elbow, shoulder, ankle and knee. Data available for power Doppler *n* = 149 for MCP 3–4, PIP 1–5 and wrist; *n* = 148 for MTP 2–3; *n* = 147 for MTP 4–5; *n* = 107 for elbow, shoulder, ankle and knee. *P ≤0.05, (Fisher’s exact test). GS: grayscale.

All joints apart from MTP 4, shoulder, ankle and knee had a higher proportion of GS and power Doppler positivity in the persistent arthritis group compared with the resolving arthritis group (Fig. 1). The greatest differences in proportion between persistent and resolving arthritis were MCP 2 GS (Δ37.7%) and MCP 3 power Doppler (Δ42.2%). On univariate logistic regression analysis, MCP 1–5, PIP 1–5, MTP 2, 3 and 5, wrist and elbow joint GS US were predictors of persistent arthritis. This was true for both GS and power Doppler variables.

In the seronegative group, MCP 1–5, PIP 1–3, MTP 3, wrist and elbow had a higher proportion of GS US pathology in the persistent arthritis group compared with the resolving arthritis group. (Supplementary Fig. S1, available at *Rheumatology* online). On univariate logistic regression analysis of the seronegative patients, these GS US variables were also predictors of seronegative persistent arthritis (Supplementary Table S9, available at *Rheumatology* online).

In the seronegative group, MCP 1–4 power Doppler, PIP 1–2 power Doppler, MTP 2 power Doppler, wrist and elbow power Doppler were more prevalent in the persistent arthritis *vs* the resolving arthritis group (Supplementary Fig. S1, available at *Rheumatology* online). On univariate logistic regression analysis, the same variables with the addition of MCP 1 power Doppler US were predictors for seronegative persistent arthritis. Univariate logistic regression analyses of joint US variables for all patients are shown in Table 2 and for seronegative patients in Supplementary Tables S9 and S10, available at *Rheumatology* online.

**Table 2:** Univariate analyses of clinical, serological and US variables in the prediction of persistent arthritis

Clinical and serological variables					
Clinical variables	*P* value	Odds ratio	95% CI		Available cases
Age ≥ 60 years*	0.010	2.792	1.284	6.071	150
Female	0.702	0.875	0.441	1.734	150
Tender joint count: 0–1 joint	Ref				
Tender joint count: 2–5 joints	0.100	2.414	0.845	6.897	147
Tender joint count: ≥ 6 joints*	0.006	3.900	1.492	10.196	
Swollen joint count: 0–1 joint	Ref				
Swollen joint count: 2–5 joints	0.322	1.603	0.630	4.082	150
Swollen joint count: ≥ 6 joints*	0.005	4.167	1.542	11.258	
Mode of onset					
Acute	Ref				
Insidious	0.140	1.860	0.816	4.240	137
Symptom duration ≥ 6 weeks*	0.015	2.355	1.178	4.708	150
Early morning stiffness duration ≥60 min*	0.000	4.133	2.021	8.452	150

Serological variables	*P* value	Odds ratio	95% CI		Available cases
Abnormal CRP	0.053	2.056	0.991	4.263	148
Abnormal ESR	0.437	1.318	0.657	2.642	149
ACPA negative	Ref				
ACPA low positivity^a^	NA	NA	NA	NA	150
ACPA high positivity*	0.002	5.875	1.945	17.747	
RF negative	Ref				
RF low positivity	0.015	12.966	1.658	101.380	150
RF high positivity*	0.003	6.753	1.920	23.755	

US variables					
Joint US variables (GS)	*P*	Odds ratio	95% CI		Available cases
MCP 1 GS*	0.000	3.587	1.724	7.464	150
MCP 2 GS*	0.000	5.205	2.500	10.838	149
MCP 3 GS*	0.000	4.156	2.028	8.513	149
MCP 4 GS*	0.001	4.271	1.925	9.473	149
MCP 5 GS*	0.000	5.793	2.377	14.114	149
PIP 1 GS*	0.003	6.490	1.869	22.537	149
PIP 2 GS*	0.000	7.811	2.860	21.336	149
PIP 3 GS*	0.003	3.498	1.535	7.972	149
PIP 4 GS*	0.006	4.764	1.571	14.451	149
PIP 5 GS*	0.006	8.108	1.836	35.810	149
MTP 2 GS*	0.010	2.550	1.249	5.207	149
MTP 3 GS*	0.000	5.631	2.197	14.430	149
MTP 4 GS	0.091	1.970	0.897	4.325	148
MTP 5 GS*	0.009	4.373	1.436	13.319	148
Wrist GS*	0.000	3.683	1.802	7.527	149
Shoulder GS	0.164	2.353	0.705	7.851	107
Elbow GS*	0.010	3.091	1.306	7.313	107
Ankle GS	0.973	1.013	0.466	2.205	107
Knee GS	0.392	1.468	0.610	3.534	107

Joint US variables (power Doppler)	*P*	Odds ratio	95% CI		Available cases
MCP 1 power Doppler*	0.001	4.018	1.813	8.903	150
MCP 2 power Doppler*	0.000	4.798	2.317	9.939	150
MCP 3 power Doppler*	0.000	6.845	3.118	15.026	149
MCP 4 power Doppler*	0.001	4.714	1.932	11.506	149
MCP 5 power Doppler*	0.008	5.405	1.546	18.894	150
PIP 1 power Doppler*	0.009	5.293	1.513	18.513	149
PIP 2 power Doppler*	0.000	8.830	2.950	26.429	149
PIP 3 power Doppler*	0.008	3.359	1.367	8.253	149
PIP 4 power Doppler*	0.009	5.293	1.513	18.513	149
PIP 5 power Doppler*	0.014	6.462	1.450	28.794	149
MTP 2 power Doppler*	0.013	13.364	1.741	102.550	148
MTP 3 power Doppler*	0.037	8.855	1.135	69.120	148
MTP 4 power Doppler	0.110	2.866	0.789	10.415	147
MTP 5 power Doppler*	0.015	3.993	1.305	12.219	147
Wrist power Doppler*	0.000	3.683	1.802	7.527	149
Shoulder power Doppler^b^	NA	NA	NA	NA	107
Elbow power Doppler*	0.011	3.642	1.337	9.921	107
Ankle power Doppler	0.746	0.875	0.390	1.962	107
Knee power Doppler	0.636	0.830	0.384	1.794	107

Tendon compartment (GS)	*P*	OR	95% CI		Available cases
Shoulder biceps GS	0.102	2.000	0.872	4.587	107
Ankle extensor GS	0.924	1.056	0.347	3.213	107
Ankle peroneal GS	0.083	2.857	0.872	9.364	107
Ankle posterior Tibial GS	0.084	2.108	0.904	4.917	107
Wrist flexor GS*	0.031	5.460	1.166	25.576	107
Wrist extensor GS*	0.020	2.533	1.158	5.544	113
Digit flexor GS*	0.000	8.000	2.807	22.803	111
Wrist ECU GS*	0.003	3.875	1.574	9.540	113
Wrist EDM GS	0.172	3.055	0.616	15.140	107
Wrist EDC/EIP GS	0.751	1.157	0.468	2.861	107
Wrist EPL GS	0.169	4.526	0.525	39.002	107
Wrist ECRL/ECRB GS	0.086	3.962	0.823	19.069	107
Wrist APL/EPB GS	0.169	4.526	0.525	39.002	107

Tendon compartment (power Doppler)	*P*	OR	95% CI		Available cases
Shoulder biceps power Doppler	0.181	1.953	0.733	5.209	107
Ankle extensor power Doppler	0.887	0.921	0.296	2.870	107
Ankle peroneal power Doppler	0.083	2.857	0.872	9.364	107
Ankle posterior tibial power Doppler*	0.030	2.769	1.104	6.945	107
Wrist flexor power Doppler*	0.023	11.180	1.405	88.990	107
Wrist extensor power Doppler*	0.020	2.533	1.158	5.544	113
Digit flexor power Doppler*	0.000	9.647	3.102	29.999	111
Wrist ECU power Doppler*	0.003	3.875	1.574	9.540	113
Wrist EDM power Doppler	0.172	3.055	0.616	15.140	107
Wrist EDC/EIP power Doppler	0.751	1.157	0.468	2.861	107
Wrist EPL power Doppler	0.169	4.526	0.525	39.002	107
Wrist ECRL/ECRB power Doppler	0.121	3.500	0.718	17.066	107
Wrist APL/EPB power Doppler	0.169	4.526	0.525	39.002	107

Univariate analyses of clinical serological and US variables at baseline in the prediction of persistent arthritis for all patients. GS grading ≥ 1; power Doppler grading ≥ 1; US pathology was present in at least unilateral joint.

a There were no patients with low-positive ACPA in the resolving arthritis group.

b There were no patients with shoulder power Doppler positive in the resolving arthritis group.

* Denotes statistical significance at *P<*0.05 level. GS: grayscale. APL: abductor pollicis longus; EPB: extensor pollicis brevis; ECRL: extensor carpi radialis longus; ECRB: extensor carpi radialis brevis; EPL: extensor pollicis longus; EDC: extensor digitorum communis; EIP: extensor indicis propius; EDM: extensor digit minimi; ECU: extensor carpi ulnaris.

### Distribution and univariate logistic regression analysis of tendon US abnormalities

**Fig. 2:**
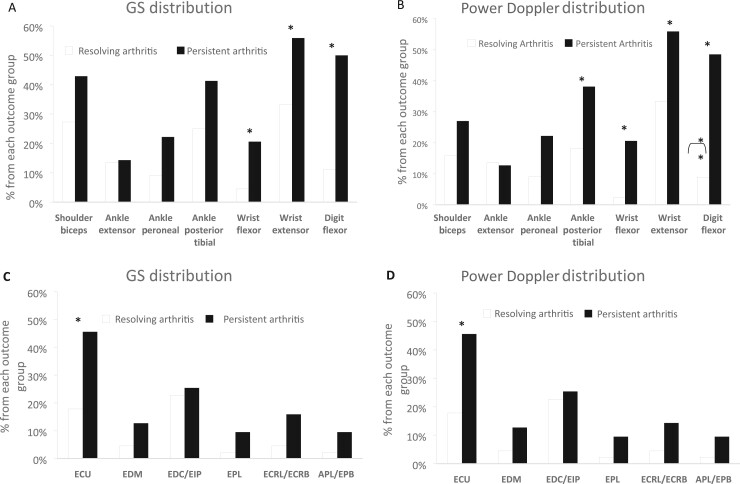
Distribution of US pathology by tendon region and wrist tendon compartment for all patients Each bar represents the proportion of patients’ US-defined tenosynovitis involvement according to tendon region individual (GS: 2A and power Doppler: 2B) and wrist extensor compartments (GS: 2C and power Doppler: 2D). Data available *n* = 113 for wrist extensor; *n* = 111 for digit flexor; *n* = 107 for shoulder biceps, ankle extensor, ankle peroneal, ankle posterior tibial and wrist flexor; *n* = 113 for ECU; *n* = 107 for EDM, EDC/EIP, EPL, ECRL/ECRB and APL/EPB. *P≤0.05, (Fisher’s exact test). APL: abductor pollicis longus; ECRB: extensor carpi radialis brevis; ECRL: extensor carpi radialis longus; ECU: extensor carpi ulnaris; EDC: extensor digitorum communis; EDM: extensor digiti minimi; EIP: extensor indicis propius; EPB: extensor pollicis brevis; EPL: extensor pollicis longus.

The prevalence of wrist flexor, wrist extensor and digit flexor TS (as assessed by both GS and power Doppler) was higher in persistent arthritis patients compared with resolving arthritis patients. This was true for both GS and power Doppler tendon US pathology (Fig. 2A, B). On univariate logistic regression analysis, the same power Doppler and GS tendon variables were predictors of persistent arthritis development (Table 2).

In the seronegative group, wrist flexor and digit flexor TS GS and power Doppler abnormalities were more likely to be present in the persistent arthritis group compared with the resolving arthritis group (Supplementary Fig. S2, available at *Rheumatology* online). On univariate logistic regression analysis, the same variables were predictors of seronegative persistent arthritis. The distribution of tendon region involvement by prognostic outcome group is shown in Fig. 2A and B and Supplementary Fig. S2, available at *Rheumatology* online for all patients and seronegative patients, respectively. Univariate logistic regression analyses of tendon US variables for all patients are shown in Table 2 and for seronegative patients in Supplementary Tables S11 and S12, available at *Rheumatology* online.

Among the six wrist extensor tendon compartments, GS and power Doppler abnormalities of the ECU tendon compartment were more likely to be present in persistent arthritis than resolving arthritis patients (Fig. 2C and D). Abnormalities of the ECU tendon compartment were also a predictor of persistent arthritis development on univariate logistic regression analysis. In seronegative patients, there was no statistical difference between the two outcome groups in any of the six wrist extensor compartments (Supplementary Fig. S3, available at *Rheumatology* online). The US pathology distribution of individual wrist compartments is shown in Fig. 2C and D and Supplementary Fig. S3, available at *Rheumatology* online for all patients and for seronegative patients, respectively.

### Univariate logistic regression analyses of clinical and serological variables

In the overall cohort, age >60 years, tender or swollen joint count of at least six joints, symptom duration of 6 weeks or more, early morning stiffness duration of at least 60 min, RF- and ACPA-high positivity were all significantly associated with the development of persistent arthritis on univariate analyses (Table 2).

For seronegative patients, age >60 years, tender joint count of at least six joints, symptom duration of at least 6 weeks and early morning stiffness at least 60 min were associated with persistent arthritis (Supplementary Table S13, available at *Rheumatology* online). Univariate analyses of clinical and serological variables are listed in Table 2 for all patients and Supplementary Table S13, available at *Rheumatology* online for seronegative patients.

### Principal component analysis

Next, statistically significant variables from the univariate logistic regression analysis were included in PCA analyses to identify the variables that accounted for the largest proportion of the variance observed. In particular, we wished to test the hypothesis that US-defined joint and tendon variables would cluster in separate components, indicating non-correlation.

PCA is a statistical analysis that can be used to reduce the overall dataset to a more manageable size, while retaining as much of the original information as possible [28]. In this study, we used PCA to identify the variables that clustered with each other and thus provided redundant information. Two PCA analyses were performed, one for clinical and serological variables (Supplementary Table S14, available at *Rheumatology* online) and one for joint and tendon US variables (Supplementary Table S15, available at *Rheumatology* online). We conducted two separate PCAs as we were interested in identifying which among the US variables clustered, or co-existed, within the same subgroup of patients, with a view to reducing the number of areas requiring scanning in each patient.

In the PCA, the number of components extracted was based on eigenvalues with a cut-off of one, and the rotation method adopted was according to the Varimax criteria with Kaiser normalization. The rotated factor loadings for each clinical, serological and US variable of the PCA are shown in Supplementary Tables S14 and S15, available at *Rheumatology* online. Three components were extracted from the clinical and serological PCA, while 10 components were extracted from the joint and tendon US PCA. Table 3 lists the clinical, serological and US variables clustered within the same PCA analysis component. The proportion of variance explained for each component is also listed. It was found that 67.5% of the variance observed could be explained by the three components from the clinical and serological PCA. In the US PCA, 80% of the variance observed was accounted for by the 10 components of the US variables PCA.

**Table 3:** Summary of principal component analysis variables

PCA of clinical and serological variables			
Components	1	2	3
Variables	Swollen joint count 66 Tender joint count 68 Early morning stiffness duration ≥60 min	RF ACPA	Symptom duration ≥6 weeks Age ≥60 years
% of variance explained	30.22	21.60	15.70
Cumulative of variance explained	67.52		

PCA of US variables										
Components	1	2	3	4	5	6	7	8	9	10
Variables	MCP 1	PIP 2	MTP 2	PIP 4	Digit flexor tendon	PIP 1	MTP 5	Wrist ECU	Wrist joint	Elbow
	MCP 2	PIP 3	MTP 3	PIP 5						
	MCP 3	PIP 4		MCP 5						
	MCP 4	PIP 5								
% of variance explained	35.99	8.56	6.30	5.93	5.07	4.51	4.11	3.56	3.19	2.85
Cumulative of variance explained	80.07									

ECU: extensor carpi ulnaris; PCA: principal component analysis.

The tendon and joint US variables were clustered separately, supporting our hypothesis. Notably, wrist ECU and wrist synovium were clustered separately in components 8 and 9, respectively. Components 1, 2 and 3 contained MCP, PIP and MTP joints, respectively. The largest variance explained from an individual component was from component 1, which contained the MCP joint variables.

### Multivariate logistic regression

Subsequently, a multivariate logistic regression model was developed using the variables identified by PCA. The variable with the highest loading factor from each component was extracted and made available as an independent variable in a forward stepwise multivariate logistic regression analysis, with persistent arthritis outcome at 18 months entered as the dependent variable. The variables included as independent variables in the multivariate logistic regression are listed in Supplementary Table S16, available at *Rheumatology* online.

The multivariate logistic regression analysis identified RF high-positivity [odds ratio (OR): 7.046], wrist power Doppler (OR: 4.391), MTP2 power Doppler (OR: 11.476) and digit flexor GS (OR: 6.586) as the variables that formed the final model for persistent arthritis prediction, with a Nagelkerke *R*^2^ value of 0.492. Removing the digit flexor tendon variable in this multivariate regression model resulted in Nagelkerke *R*^2^ falling from 0.492 to 0.327 (Table 4). Therefore, the digit flexor tendon variable alone contributed 16.5% of the predictive power of this model for persistent arthritis in our cohort, after taking into account the presence of RF and wrist power Doppler and MTP2 power Doppler variables.

**Table 4:** Final multivariate logistic regression model for the prediction of persistent arthritis

Variable	Odds ratio	95% CI	*P*-value	Nagelkerke *R*^2^
**Model 1**				
RF				
Negative	Reference		0.022	0.492
Low positive	8.270	0.821–83.279	0.073	
High positive	7.046	1.275–38.943	0.025	
Wrist power Doppler	4.391	1.565–12.324	0.005	
MTP2 power Doppler	11.476	1.135–115.990	0.039	
Digit flexor tendon GS positive	6.586	1.967–22.053	0.002	
**Model 2**				
RF				
Negative	Reference		0.004	0.327
Low positive	12.891	1.596–104.104	0.016	
High positive	5.245	1.407–19.554	0.014	
Wrist power Doppler	3.073	1.389–6.798	0.006	
MTP2 power Doppler	11.913	1.489–95.294	0.020	

Forward stepwise multivariate logistic regression analysis, with persistent arthritis outcome at 18 months entered as the dependent variable and variables from Supplementary Table S16, available at *Rheumatology* online as the independent variables. Model 2 shows the effect on the Nagelkerke *R*^2^ value when digit flexor GS tendon variable was removed from the logistic regression model. GS: grayscale.

### PCA and multivariate logistic regression analysis for seronegative patients

A similar PCA analysis was performed for the seronegative cohort; one for clinical and serological variables **(**Supplementary Table S17, available at *Rheumatology* online), and one for tendon and joint US variables (Supplementary Table S18, available at *Rheumatology* online). Two components were extracted from the clinical and serological PCA, while seven components were extracted from the joint and tendon US PCA.

Supplementary Table S19, available at *Rheumatology* online, lists the clinical, serological and US variables clustered within the same PCA component. The proportion of variance explained for each component is also listed. It was found that 63.2% of the variance observed could be explained by the two components from the clinical and serological PCA of seronegative patients. In the US PCA of seronegative patients, 80.5% of the variance observed was explained by the seven components of the US variables PCA.

Similar to the PCA analysis of the overall cohort, in seronegative disease, the tendon and synovial variables clustered under different components (tendon variables within components 4 and 5—the remaining components were joint US variables). Wrist flexor tendon and wrist synovium variables were in two separate components. In addition, the digit flexor tendon was separate from the MCP and PIP synovial components. The variable with the highest loading factor from each component was extracted and made available as an independent variable in a forward stepwise multivariate logistic regression analysis, with seronegative persistent arthritis outcome at 18 months as the dependent variable. The variables included as independent variables in the multivariate logistic regression are listed in Supplementary Table S20, available at *Rheumatology* online. The resulting logistic regression showed that PIP2 GS and digit flexor tendon GS were independent predictors of seronegative persistent arthritis, with a Nagelkerke *R*^2^ value of 0.304 (Supplementary Table S21, available at *Rheumatology* online).

## Discussion

This is the first study to show that US-defined TS, specifically digit flexor TS, is an independent predictor of arthritis persistence in an inception cohort of patients with early arthritis. The predictive value of digit flexor TS remained even after taking into account synovial US, and clinical and serological variables. This was also true for persistent arthritis patients who were RF/ACPA negative. This work follows on from our previous report that US-defined TS predicts RA development in patients with early arthritis [17]. In this work, we are addressing an important evidence gap, which is to identify whether US markers have a role in predicting persistent arthritis development in those with no measurable ACPA/RF antibodies. This is the reason why we conducted the analysis of the seronegative subgroup. A large study of 11 237 tendons (bilateral digit flexor 1–5 and ECU tendon) from 939 healthy individuals concluded that tendon abnormalities identified by US can be regarded as markers of inflammation, regardless of age group and level of physical activity [29]. It was found that 98% of these tendons were graded 0 for GS TS, power Doppler TS and tenosynovial effusion. Furthermore, 99% (931/939) of healthy individuals had no power Doppler TS in any tendons. In this study, we demonstrated that GS digit flexor TS, even in the earliest disease phase, within 3 months of symptom onset, predicts the development of persistent arthritis in a cohort of patients with early arthritis.

To date, studies assessing the predictive value of US have focused on data from the assessment of small joint synovia rather than tendons [10–13]. In a large cohort of patients with early arthritis (*n* = 831), US data facilitated in the identification of those whose arthritis persisted (including in the ACPA-negative group). Sonographers’ impressions of the scanning data (classified as definitely inflammatory, possibly inflammatory, non-inflammatory) of the symptomatic wrist, MCP and PIP joints improved the area under the curve from 0.81 to 0.90. In that study, however, tendons were not included in the scanning algorithm. The investigators scanned wrist, MCP 2–3, PIP 2–3 and MTP 2–5 joints of the most symptomatic side (or dominant side if equally symptomatic). The sum of the GS and power Doppler scores was strongly associated with disease persistence [11]. In our study, we scanned a wider range of synovium joints and tendons, including the large joints and tendons (shoulder, elbow, ankle and knees) as well as all the small joints (MCP 1–5, PIP 1–5, wrist and MTP 1–5) and tendons (wrist flexor and extensor compartments and digit flexor tendons 1–5).

In a cohort of patients (*n* = 50) with musculoskeletal symptoms of <12 weeks and without RF or ACPA autoantibodies, US features of MCP or wrist synovium such as grayscale US grade 3, presence of power Doppler and at least one US erosion increased the probability of developing persistent arthritis. However, that study did not assess the independent predictive value of joint and tendon US separately, as the small sample size precluded logistic regression analysis [30].

Digit flexor TS in patients with RA is widely reported [31–34]. However, digit flexor TS in non-RA inflammatory arthritis is less well recognized. Olivieri *et al.* reported that clinical dactylitis corresponded to flexor TS on MRI and US imaging [35]. These findings were subsequently replicated by two US studies in patients with PsA [36, 37]. Furthermore, there was no significant difference in the frequency of hand TS between early RA and early PsA in an MRI study reported by Narvaez *et al.* [38], indicating that hand TS may be an equally important early marker of inflammatory joint involvement in both early RA and early PsA.

In addition, US studies have shown that synovitis and/or flexor TS alongside soft tissue thickening and oedema were the elementary US lesions in dactylitis [39]. In this work, we did not record the presence of clinical dactylitis in individual joints. However, the proportion of patients who have conditions associated with dactylitis, such as PsA, AS, peripheral SpA and reactive arthritis was low (18 out of 150 patients); therefore, this is unlikely to have affected the overall outcome of this study.

A common challenge in US prediction studies is identifying the potential joint and tendon areas that provide the maximal predictive ability for a specified outcome. We used PCA techniques to identify redundant US variables. One of our significant findings is that *tendon* US variables are not redundant in relation to the neighbouring *joint* US variables. These findings highlight that tendon US variables provide predictive data independent from that of joint US variables.

A strength of this study is the extensive range of joint and tendon regions assessed. Furthermore, data for the ACPA/RF-negative patients were analysed separately, which revealed that the predictive value for digit flexor tendons remains important in this subgroup.

A limitation of this study was that the individual flexor tendons (i.e. digit flexor tendons 1–5) were not scored. In clinical practice, scanning specific digit flexor tendons could reduce scanning time. Future work should identify the specific digit flexor tendons that contribute to persistent arthritis prediction.

In conclusion, US defined digit flexor tendon TS is an independent predictor of persistent arthritis—even after taking into account conventional synovial US, and clinical and serological variables. Investigators designing scanning panels and predictive algorithms for imaging studies for persistent arthritis development should consider including the digit flexor tendon as a candidate variable.

## Supplementary Material

keac199_Supplementary_Data

## Data Availability

The data underlying this article will be shared on reasonable request to the corresponding author.
