# Supplementary material for: The value of ultrasound-defined tenosynovitis and synovitis in the prediction of persistent arthritis
Source: Rheumatology (Oxford). 2022 Apr 12;62(3):1057–68. doi: 10.1093/rheumatology/keac199 (PMC9977123; doi:10.1093/rheumatology/keac199)
Supplement: keac199_Supplementary_Data [file keac199_supplementary_data.docx]

Contents

[Supplementary table S1 Synovial intra-articular recesses and periarticular sites evaluated by ultrasound 3](#_Toc96286708)

[Supplementary table S2 Tendon compartments evaluated by ultrasound 4](#_Toc96286709)

[Supplementary table S3 Intra-observer reliability of joint ultrasound assessment 5](#_Toc96286710)

[Supplementary table S4 Intra-observer reliability of tendon ultrasound assessment 5](#_Toc96286711)

[Supplementary table S5 Inter-observer reliability of joint ultrasound assessment 6](#_Toc96286712)

[Supplementary table S6 Classification of variables for the logistic regression analysis 6](#_Toc96286713)

[Supplementary table S7 Baseline characteristics according to outcome group for seronegative patients (n=98) 7](#_Toc96286714)

[Supplementary table S8A Final diagnoses according to outcome groups for all patients 8](#_Toc96286715)

[Supplementary table S8B Final diagnoses according to outcome group for seronegative patients 8](#_Toc96286716)

[Supplementary figure S1 Distribution of joint ultrasound pathology for seronegative patients, n=98 (GS and PD). 9](#_Toc96286717)

[Supplementary table S9 Univariate analysis of joint GS US variables at baseline in the prediction of persistent arthritis for seronegative patients (n=98) 10](#_Toc96286718)

[Supplementary table S10 Univariate analysis of joint PD US variables at baseline in the prediction of persistent arthritis for seronegative patients (n=98) 11](#_Toc96286719)

[Supplementary figure S2 Distribution of tendon compartment ultrasound pathology for seronegative patients, n=98 (GS and PD). 12](#_Toc96286720)

[Supplementary table S11 Univariate analysis of tendon compartment GS tenosynovitis at baseline in the prediction of persistent arthritis for seronegative patients (n=98) 13](#_Toc96286721)

[Supplementary table S12 Univariate analysis of tendon compartment PD tenosynovitis at baseline in the prediction of persistent arthritis for seronegative patients (n=98) 13](#_Toc96286722)

[Supplementary figure S3 Distribution of individual wrist tendon compartment US pathology, n=98 (GS and PD) 14](#_Toc96286723)

[Supplementary table S13 Univariate analyses of clinical and serological variables at baseline in the prediction of persistent arthritis for seronegative patients (n=98) 15](#_Toc96286724)

[Supplementary table S14 Principal component analysis of clinical and serological variables for all patients 16](#_Toc96286725)

[Supplementary table S15 Principal component analysis of US variables for all patients 17](#_Toc96286726)

[Supplementary table S16 Variables included in the logistic regression model for all patients 19](#_Toc96286727)

[Supplementary table 17 Principal component analysis of clinical and serological variables for seronegative patients 19](#_Toc96286728)

[Supplementary table S18 Principal component analysis of ultrasound variables for seronegative patients 20](#_Toc96286729)

[Supplementary table 19 Summary of PCA variables for seronegative patients 21](#_Toc96286730)

[Supplementary table S20 Variables included in the forward stepwise logistic regression model for seronegative patients 22](#_Toc96286731)

[Supplementary table 21 Logistic regression model for seronegative patients 22](#_Toc96286732)

# Supplementary table S1 Synovial intra-articular recesses and periarticular sites evaluated by ultrasound

| **Joint*** | **Recess** |
| --- | --- |
| **MCP (1-5), PIP (1-5), MTP (2-5)** | Multi-planar scanning of dorsal recesses |
|  | Radial aspect of MCP 1, 2  Lateral aspect of MTP5 |
| **Wrist** | Intercarpal recesses |
|  | Radiocarpal recesses |
|  | Ulnarcarpal recesses |
|  | Volar carpal recesses |
| **Elbow** | Anterior recess |
|  | Humeroradial joint |
|  | Humeroulnar joint |
|  | Posterior recess |
| **Shoulder** | Posterior glenohumeral recess |
| **Knee** | Suprapatellar recess |
|  | Medial parapatellar recess |
|  | Lateral parapatellar recess |
|  | Medial femorotibial joint line |
|  | Lateral femorotibial joint line |
| **Ankle** | Anterior tibiotalar recess |
|  | Medial tibiotalar recess |
|  | Lateral tibiotalar recess |

MCP: metacarpophalangeal joint; MTP: metatarsophalangeal joint; PIP: proximal interphalangeal joint.

# Supplementary table S2 Tendon compartments evaluated by ultrasound

| Joint region | Tendon Compartment |
| --- | --- |
| Hand | Flexor tendons (1-5) |
| Wrist | Wrist flexor tendon |
|  | Extensor Compartment 1 (i.e. APL and EPB) |
|  | Extensor Compartment 2 (i.e. ECRL and ECRB) |
|  | Extensor Compartment 3 (i.e. EPL) |
|  | Extensor Compartment 4 (i.e. EDC and EIP) |
|  | Extensor Compartment 5 (i.e. EDM) |
|  | Extensor Compartment 6 (i.e. ECU) |
| Shoulder | Biceps tendon |
| Ankle | Anterior extensor compartment |
|  | Peroneal tendons |
|  | Posterior tibialis tendon |

APL: abductor pollicis longus; EPB: extensor pollicis brevis; ECRL: extensor carpi radialis longus; ECRB: extensor carpi radialis brevis; EPL: extensor pollicis longus; EDC: extensor digitorum communis; EIP: extensor indicis propius; EDM: extensor digiti minimi; ECU: extensor carpi ulnaris.

# Supplementary table S3 Intra-observer reliability of joint ultrasound assessment

| **Joint assessment** | **Greyscale** | **Power Doppler** |
| --- | --- | --- |
| **Overall** | **0.83** | **0.87** |
| PIP | 0.85 | 0.91 |
| MCP | 0.85 | 0.91 |
| Wrist | 0.77 | 0.81 |
| Elbow | 0.81 | 0.82 |
| Shoulder | 0.86 | 0.65 |
| Knee | 0.77 | 0.70 |
| Ankle | 0.77 | 0.75 |
| MTP | 0.76 | 0.88 |

A kappa value of 0-0.2 was considered poor, 0.21-0.40 fair, 0.41-0.6 moderate, 0.61-0.8 good, and 0.81 to 1 excellent.

# Supplementary table S4 Intra-observer reliability of tendon ultrasound assessment

| **Tendon assessment** | **Greyscale** | **Power Doppler** |
| --- | --- | --- |
| **Overall** | **0.96** | **0.95** |
| Digit | 0.99 | 0.99 |
| Wrist | 0.97 | 0.95 |
| Shoulder | 0.94 | 0.86 |
| Ankle | 0.96 | 0.95 |
| **Tendon Compartments** | **Greyscale** | **Power Doppler** |
| Digit flexor | 0.99 | 0.99 |
| Wrist flexor | 0.97 | 0.97 |
| Wrist extensor | 0.97 | 0.94 |
| Shoulder | 0.94 | 0.86 |

# Supplementary table S5 Inter-observer reliability of joint ultrasound assessment

| **Joint assessment** | **Greyscale** | **Power Doppler** |
| --- | --- | --- |
| **Overall** | **0.568** | **1.00** |
| MCP | 1.00 | 1.00 |
| PIP | 0.64 | 1.00 |
| Wrist | 0.78 | 1.00 |
| MTP | 0.40 | 1.00 |
| Ankle anterior | 0.97 | 0.96 |
| Ankle posteromedial | 0.99 | 0.99 |
| Ankle peroneal | 0.88 | 0.88 |

A kappa value of 0-0.2 was considered poor, 0.21-0.40 fair, 0.41-0.6 moderate, 0.61-0.8 good, and 0.81 to 1 excellent.

# Supplementary table S6 Classification of variables for the logistic regression analysis

| **Variables** | **Category** |
| --- | --- |
| Sex | Male*/female |
| Age | < 60 years*/ ≥60 years |
| ESR | Normal*/abnormal (by local standards) |
| CRP | Normal*/abnormal (by local standards) |
| Tender joint count-68 | 0-1*, 2–5, 6–68 |
| Swollen joint count-66 | 0-1*, 2–5, 6–66 |
| Early morning stiffness | <60 minutes* / ≥60 minutes |
| Rheumatoid factor (RF) | Normal* / low-positive^a^/high-positive^b^ |
| Anti-cyclic citrullinated peptide (ACPA) | Normal* / low-positive^c^/high-positive^d^ |
| Mode of onset | Acute*/Insidious |

*Reference category. ^a^ RF > 20 IU/mL, ^b^ RF > 60 IU/mL, ^c^ACPA >7 EU/ml, ^d^ACPA >21 EU/ml.

# Supplementary table S7 Baseline characteristics according to outcome group for seronegative patients (n=98)

|  | Resolving Inflammatory Arthritis | Persistent Inflammatory Arthritis | p |
| --- | --- | --- | --- |
| N | 44 | 54 |  |
| Age, years | 44 (34-58) | 56 (39-69) | 0.060^b^ |
| Female, n (%) | 26 (59.1) | 29 (53.7) | 0.684^a^ |
| Symptom duration, weeks | 5 (3-8) | 7 (5-10) | 0.004^b^ |
| Early morning stiffness*, minutes | 30 (6-83) | 90 (30-240) | 0.001^b^ |
| ACPA, n (%)  Negative | 44 (100.0) | 54 (100.0) | ^NA^ |
| RF, n (%)  Negative | 44 (100.0) | 54 (100.0) | ^NA^ |
| Mode of onset^φ^, n (%)  Acute  Insidious | 33 (82.5)  7 (17.5) | 36 (70.6)  15 (29.4) | 0.224^a^ |
| NSAID use, n (%) | 27 (61.4) | 31 (57.4) | 0.837^a^ |
| CRP, mg/l | 10 (2- 28) | 13 (3-32) | 0.456^b^ |
| ESR*, mm/h | 18 (5-36) | 20 (10 – 49) | 0.384^b^ |
| Tender joint count of 68** | 5 (2-8) | 8 (4-17) | 0.002^b^ |
| Swollen joint count of 66 | 2 (2-6) | 6 (2-12) | 0.006^b^ |
| Tender joint count of 28 | 2 (1-5) | 6 (2-13) | 0.001^b^ |
| Swollen joint count of 28 | 2 (1-4) | 5 (1-11) | <0.001^b^ |
| DAS-28 CRP | 3.45 (2.99 -4.58) | 4.70 (3.48 – 5.50) | 0.001^b^ |
| DAS-28 ESR^*^ | 3.84 (3.01 – 4.47) | 4.81 (3.69-6.06) | 0.001^b^ |

All variables are shown as median (IQR) unless otherwise specified. ^a^ Fisher’s exact test, ^b^ Mann-Whitney test, *n= 97, **n=95, ^φ^n=91, , ACPA, anti-cyclic citrullinated peptide antibody; CRP, C-reactive protein; DAS28, disease activity score in 28 joints; ESR, erythrocyte sedimentation rate NSAID, non-steroidal anti-inflammatory drug; RF, rheumatoid factor.

# Supplementary table S8A Final diagnoses according to outcome groups for all patients

| Diagnosis | Resolving Inflammatory  Arthritis,  n (%) | Persistent Inflammatory Arthritis,  n (%) | Total,  n |
| --- | --- | --- | --- |
| Rheumatoid Arthritis | 12 (23.5) | 73 (73.7) | 85 |
| Psoriatic Arthritis | 2 (3.9) | 12** (12.1) | 14 |
| Unclassified Arthritis | 23 (45.1) | 7 (7.1) | 30 |
| SLE | 0 (0) | 3 (3.0) | 3 |
| Sarcoidosis | 0 (0) | 2 (2.0) | 2 |
| Palindromic Arthritis | 1 (2.0) | 0 (0) | 1 |
| Ankylosing Spondylitis | 0 (0) | 1 (1.0) | 1 |
| Peripheral SpA | 0 (0) | 1 (1.0) | 1 |
| Reactive Arthritis | 2 (4.0) | 0 (0) | 2 |
| Parvovirus Arthritis | 5* (9.8) | 0 (0) | 5 |
| Gout | 3 (5.9) | 0 (0) | 3 |
| Pseudo-gout | 2 (3.9) | 0 (0) | 2 |
| Septic Arthritis | 1 (2) | 0 (0) | 1 |
|  | **51 (100)** | **99 (100)** | **150** |
| Supplementary table S8B Final diagnoses according to outcome group for seronegative patients | | | |
| Diagnosis | **Resolving Inflammatory**  **Arthritis,**  **n (%)** | **Persistent Inflammatory Arthritis,**  **n (%)** | **Total,**  **n** |
| Rheumatoid Arthritis | 9 (20.5) | 33 (61.1) | 42 |
| Psoriatic Arthritis | 2 (4.5) | 9 (16.7) | 11 |
| Unclassified Arthritis | 21 (47.7) | 6 (11.1) | 27 |
| SLE | 0 (0) | 3 (5.6) | 3 |
| Sarcoidosis | 0 (0) | 2 (3.7) | 2 |
| Ankylosing Spondylitis | 0 (0) | 1 (1.9) | 1 |
| Reactive Arthritis | 2 (4.5) | 0 (0) | 2 |
| Parvovirus Arthritis | 5 (11.4) | 0 (0) | 5 |
| Gout | 2 (4.5) | 0 (0) | 2 |
| Pseudo-gout | 2 (4.5) | 0 (0) | 2 |
| Septic Arthritis | 1 (2.3) | 0 (0) | 1 |
| Total, n | **44** | **54** | **98** |

*One patient with parvovirus arthritis was on MTX for a different indication at final follow-up.

**One patient with PsA had an initial diagnosis of reactive arthritis secondary to campylobacter at baseline.

# Supplementary figure S1 Distribution of joint ultrasound pathology for seronegative patients, n=98 (GS and PD).

|  |
| --- |
|  |
| *p≤0.05, ** p≤0.01, *** p≤0.001 (Fisher’s exact test). Each bar represents the proportion of patients with ultrasound-defined synovitis involvement according to outcome groups. Data available for GS, n=97 for MCP2-3, PIP1-5, MTP2-3 and wrist; n=96 for MTP4-5; n=75 for elbow, shoulder, ankle and knee. Data available for PD, n=97 for MCP 3-4, PIP 1-5, MTP 2-3 and wrist; n=95 for MTP4-5, n=75 for elbow, shoulder, ankle and knee. |

# Supplementary table S9 Univariate analysis of joint GS US variables at baseline in the prediction of persistent arthritis for seronegative patients (n=98)

| **Joint US variables (GS)** | **p** | **Odds Ratio** | **95% CI** | | **Data available, n** |
| --- | --- | --- | --- | --- | --- |
| MCP 1 GS* | .028 | 2.568 | 1.109 | 5.944 | 98 |
| MCP 2 GS* | .002 | 3.835 | 1.644 | 8.946 | 97 |
| MCP 3 GS* | .022 | 2.621 | 1.151 | 5.966 | 97 |
| MCP 4 GS* | .004 | 3.745 | 1.509 | 9.296 | 97 |
| MCP 5 GS* | .001 | 5.241 | 1.896 | 14.489 | 97 |
| PIP 1 GS* | .022 | 4.667 | 1.244 | 17.499 | 97 |
| PIP 2 GS* | .005 | 5.293 | 1.641 | 17.067 | 97 |
| PIP 3 GS* | .033 | 2.784 | 1.084 | 7.149 | 97 |
| PIP 4 GS | .143 | 2.494 | .738 | 8.480 | 97 |
| PIP 5 GS | .082 | 4.100 | .836 | 20.096 | 97 |
| MTP 2 GS | .097 | 2.010 | .882 | 4.581 | 97 |
| MTP 3 GS* | .009 | 3.924 | 1.413 | 10.899 | 97 |
| MTP 4 GS | .448 | 1.427 | .569 | 3.577 | 96 |
| MTP 5 GS | .133 | 2.554 | .750 | 8.689 | 96 |
| Wrist GS* | .009 | 3.023 | 1.312 | 6.966 | 97 |
| Shoulder GS | .775 | .795 | .165 | 3.826 | 75 |
| Elbow GS* | .032 | 2.900 | 1.098 | 7.660 | 75 |
| Ankle GS | .316 | .625 | .250 | 1.565 | 75 |
| Knee GS | .552 | 1.375 | .481 | 3.932 | 75 |

GS: Grey scale. * denotes statistical significance at 0.05 level.

# Supplementary table S10 Univariate analysis of joint PD US variables at baseline in the prediction of persistent arthritis for seronegative patients (n=98)

| **Joint US variables (PD)** | **p-value** | **Odds Ratio** | **95% CI** | | **Available data, n** |
| --- | --- | --- | --- | --- | --- |
| MCP 1 PD* | .027 | 2.720 | 1.121 | 6.598 | 98 |
| MCP 2 PD* | .009 | 3.038 | 1.326 | 6.963 | 98 |
| MCP 3 PD* | .001 | 4.227 | 1.764 | 10.132 | 97 |
| MCP 4 PD* | .013 | 3.469 | 1.305 | 9.220 | 97 |
| MCP 5 PD | .102 | 3.106 | .798 | 12.084 | 98 |
| PIP 1 PD* | .033 | 4.228 | 1.119 | 15.966 | 97 |
| PIP 2 PD* | .013 | 4.480 | 1.379 | 14.556 | 97 |
| PIP 3 PD | .049 | 2.833 | 1.005 | 7.986 | 97 |
| PIP 4 PD | .162 | 2.667 | .675 | 10.540 | 97 |
| PIP 5 PD | .121 | 3.565 | .716 | 17.759 | 97 |
| MTP 2 PD* | .026 | 10.744 | 1.328 | 86.936 | 97 |
| MTP 3 PD | .192 | 4.286 | .481 | 38.150 | 97 |
| MTP 4 PD | .153 | 2.727 | .690 | 10.787 | 96 |
| MTP 5 PD | .195 | 2.267 | .658 | 7.819 | 96 |
| Wrist PD* | .009 | 3.023 | 1.312 | 6.966 | 97 |
| Shoulder PD | NA | NA | NA | NA | NA |
| Elbow PD* | .025 | 3.500 | 1.167 | 10.494 | 75 |
| Ankle PD | .347 | .633 | .244 | 1.642 | 75 |
| Knee PD | .283 | .605 | .241 | 1.514 | 75 |

PD: Power Doppler; * denotes statistical significance at 0.05 level.

# Supplementary figure S2 Distribution of tendon compartment ultrasound pathology for seronegative patients, n=98 (GS and PD).

|  |
| --- |
|  |
| *p≤0.05, ** p≤0.01, *** p≤0.001 (Fisher’s exact test). Each bar represents the proportion of patients with ultrasound-defined tenosynovitis involvement according to outcome groups. Data available for GS and PD, n=78 for wrist extensor and digit flexor; n=75 for shoulder biceps, ankle extensor, ankle peroneal, ankle posterior tibial and wrist flexor. |

# Supplementary table S11 Univariate analysis of tendon compartment GS tenosynovitis at baseline in the prediction of persistent arthritis for seronegative patients (n=98)

| Tendon Compartment (GS) | p | OR | 95% CI | | Available data, n |
| --- | --- | --- | --- | --- | --- |
| Shoulder Biceps GS | .090 | 2.320 | .876 | 6.145 | 75 |
| Ankle Extensor GS | .355 | .500 | .115 | 2.169 | 75 |
| Ankle Peroneal GS | .905 | 1.094 | .252 | 4.740 | 75 |
| Ankle Posterior Tibial GS | .636 | 1.276 | .465 | 3.502 | 75 |
| Wrist Flexor GS* | .045 | 5.286 | 1.040 | 26.854 | 75 |
| Wrist Extensor GS | .182 | 1.869 | .746 | 4.682 | 78 |
| Digit Flexor GS* | <.001 | 7.778 | 2.505 | 24.148 | 78 |
| ECU GS | .059 | 2.750 | .964 | 7.848 | 78 |
| EDM GS | .293 | 3.455 | .343 | 34.828 | 75 |
| EDC/EIP GS | .702 | .805 | .265 | 2.446 | 75 |
| EPL GS | .519 | 2.235 | .194 | 25.765 | 75 |
| ECRL/ECRB GS | .210 | 2.984 | .541 | 16.462 | 75 |
| APL/EPB GS | .519 | 2.235 | .194 | 25.765 | 75 |

GS: Grey scale; TS: tenosynovitis. * denotes statistical significance at 0.05 level.

# Supplementary table S12 Univariate analysis of tendon compartment PD tenosynovitis at baseline in the prediction of persistent arthritis for seronegative patients (n=98)

| Tendon Compartment (PD) | p | OR | 95% CI | | Available data, n |
| --- | --- | --- | --- | --- | --- |
| Shoulder Biceps PD | .458 | 1.524 | .501 | 4.636 | 75 |
| Ankle Extensor PD | .355 | .500 | .115 | 2.169 | 75 |
| Ankle Peroneal PD | .905 | 1.094 | .252 | 4.740 | 75 |
| Ankle Posterior Tibial PD | .313 | 1.758 | .588 | 5.260 | 75 |
| Wrist Flexor PD* | .029 | 10.857 | 1.283 | 91.856 | 75 |
| Wrist Extensor PD | .182 | 1.869 | .746 | 4.682 | 78 |
| Digit Flexor PD* | <.001 | 9.000 | 2.676 | 30.273 | 78 |
| ECU PD | .059 | 2.750 | .964 | 7.848 | 78 |
| EDM PD | .293 | 3.455 | .343 | 34.828 | 75 |
| EDC/EIP PD | .702 | .805 | .265 | 2.446 | 75 |
| EPL PD | .519 | 2.235 | .194 | 25.765 | 75 |
| ECRL/ECRB PD | .351 | 2.312 | .397 | 13.469 | 75 |
| APL/EPB PD | .519 | 2.235 | .194 | 25.765 | 75 |

*Denotes statistical significance at 0.05 level.

# Supplementary figure S3 Distribution of individual wrist tendon compartment US pathology, n=98 (GS and PD)

|  |
| --- |
|  |
| *p≤0.05, ** p≤0.01, *** p≤0.001 (Fisher’s exact test). Each bar represents the proportion of patients with ultrasound-defined tenosynovitis involvement of wrist extensor tendon compartments according to outcome groups. APL: Abductor pollicis longus; EPB: extensor pollicis brevis; ECRL: extensor carpi radialis longus; ECRB: extensor carpi radialis brevis; EPL: extensor pollicis longus; EDC: extensor digitorum communis; EIP: extensor indicis propius; EDM: extensor digit minimi; ECU: extensor carpi ulnaris. Data available for GS and PD, n=78 for ECU; n=75 for EDM, EDC/EIP, EPL, ECRL/ECRB and APL/EPB. |

# Supplementary table S13 Univariate analyses of clinical and serological variables at baseline in the prediction of persistent arthritis for seronegative patients (n=98)

| Clinical and serological variables | P value | Odds Ratio | 95% CI | | Data available |
| --- | --- | --- | --- | --- | --- |
| Age ≥ 60 years old* | .022 | 2.885 | 1.162 | 7.166 | 98 |
| Female | .593 | .803 | .359 | 1.795 | 98 |
| Tender joint count: 0-1 joint  Tender joint count: 2-5 joints  Tender joint count: ≥ 6 joints* | Ref  .087  .023 | 3.333  4.474 | .838  1.234 | 13.251  16.224 | 95 |
| Swollen joint count: 0-1 joint  Swollen joint count: 2-5 joints  Swollen joint count: ≥ 6 joints | Ref  .865  .111 | .909  2.500 | .304  .809 | 2.717  7.727 | 98 |
| Mode of onset  Acute  Insidious | Ref  .192 | 1.964 | .713 | 5.414 | 91 |
| Symptom duration ≥ 6 weeks* | .023 | 2.612 | 1.144 | 5.964 | 98 |
| Early morning stiffness duration ≥ 60 min* | .013 | 2.864 | 1.251 | 6.555 | 98 |
| Abnormal CRP | .687 | .998 | .988 | 1.008 | 98 |
| Abnormal ESR | .571 | 1.004 | .990 | 1.019 | 97 |

* Denotes statistical significance at 0.05 level.

# Supplementary table S14 Principal component analysis of clinical and serological variables for all patients

| **Variables** | **Component** | | |
| --- | --- | --- | --- |
|  | **1** | **2** | **3** |
| **Swollen joint count-66**  **(0-1, 2-5,** **≥ 6)** | .843 |  |  |
| Tender joint count-68  (0-1, 2-5, ≥ 6) | .817 |  |  |
| Early morning stiffness duration ≥ 60 minutes | .606 |  |  |
| **Rheumatoid factor**  **(negative, low positive, high positive)** |  | .934 |  |
| ACPA  (negative, low positive, high positive) |  | .929 |  |
| **Symptom duration ≥ six weeks** |  |  | .770 |
| Age ≥ 60 years |  |  | .768 |

Rotation method: Varimax with Kaiser Normalization. Factor loadings of <0.400 are suppressed to facilitate interpretation. The variable with the highest loading factor from each component is highlighted in bold. aACPA >7 EU/ml, bACPA >21 EU/ml, cRF > 20 IU/mL, d RF > 60 IU/mL.

# Supplementary table S15 Principal component analysis of US variables for all patients

| **Rotated Component Matrix** | | | | | | | | | | |
| --- | --- | --- | --- | --- | --- | --- | --- | --- | --- | --- |
|  | **Component** | | | | | | | | | |
| **US variables** | **1** | **2** | **3** | **4** | **5** | **6** | **7** | **8** | **9** | **10** |
| **MCP3 GS** | 0.792 |  |  |  |  |  |  |  |  |  |
| MCP3 PD | 0.775 |  |  |  |  |  |  |  |  |  |
| MCP2 PD | 0.772 |  |  |  |  |  |  |  |  |  |
| MCP1 GS | 0.722 |  |  |  |  |  |  |  |  |  |
| MCP2 GS | 0.712 |  |  |  |  |  |  |  |  |  |
| MCP4 GS | 0.711 |  |  |  |  |  |  |  |  |  |
| MCP1 PD | 0.698 |  |  |  |  |  |  |  |  |  |
| MCP4 PD | 0.664 |  |  |  |  |  |  |  |  |  |
| **PIP3 GS** |  | 0.843 |  |  |  |  |  |  |  |  |
| PIP3 PD |  | 0.818 |  |  |  |  |  |  |  |  |
| PIP2 GS |  | 0.752 |  |  |  |  |  |  |  |  |
| PIP2 PD |  | 0.716 |  |  |  |  |  |  |  |  |
| PIP5 GS |  | 0.646 |  |  | 0.481 |  |  |  |  |  |
| PIP5 PD |  | 0.627 |  |  | 0.484 |  |  |  |  |  |
| **Digit flexor tendon GS** |  |  | 0.808 |  |  |  |  |  |  |  |
| Digit flexor tendon PD |  |  | 0.801 |  |  |  |  |  |  |  |
| Wrist flexor tendon PD |  |  | 0.763 |  |  |  |  |  |  |  |
| Wrist flexor tendon GS |  |  | 0.761 |  |  |  |  |  |  |  |
| Ankle posterior tibial tendon PD |  |  |  |  |  |  |  |  |  |  |
| **MTP2 PD** |  |  |  | 0.761 |  |  |  |  |  |  |
| MTP3 PD |  |  |  | 0.754 |  |  |  |  |  |  |
| MTP2 GS |  |  |  | 0.687 |  |  |  |  |  |  |
| MTP3 GS |  |  |  | 0.664 |  |  |  |  |  |  |
| **MCP5 PD** |  |  |  |  | 0.717 |  |  |  |  |  |
| PIP4 PD |  | 0.466 |  |  | 0.615 |  |  |  |  |  |
| PIP4 GS |  |  |  |  | 0.571 |  |  |  |  |  |
| MCP5 GS |  |  |  |  | 0.529 |  |  |  |  |  |
| **PIP1 PD** |  |  |  |  |  | 0.818 |  |  |  |  |
| PIP1 GS |  |  |  |  |  | 0.802 |  |  |  |  |
| **MTP5 PD** |  |  |  |  |  |  | 0.870 |  |  |  |
| MTP5 GS |  |  |  |  |  |  | 0.857 |  |  |  |
| **Wrist ECU GS** |  |  |  |  |  |  |  | 0.856 |  |  |
| Wrist ECU PD |  |  |  |  |  |  |  | 0.856 |  |  |
| **Wrist PD** |  |  |  |  |  |  |  |  | 0.876 |  |
| Wrist GS |  |  |  |  |  |  |  |  | 0.876 |  |
| **Elbow GS** |  |  |  |  |  |  |  |  |  | 0.894 |
| Elbow PD |  |  |  |  |  |  |  |  |  | 0.865 |
| Rotation method: Varimax with Kaiser Normalization. Factor loadings of <0.400 are suppressed to facilitate interpretation. The variable with the highest loading factor from each component is highlighted in bold. ECU: Extensor carpi ulnaris; GS: Grey scale; PD: Power Doppler | | | | | | | | | | |

# Supplementary table S16 Variables included in the logistic regression model for all patients

| **Clinical and serological variables** | **Ultrasound variables** |
| --- | --- |
| Swollen joint–66 (3 levels)  Rheumatoid Factor (3 levels)  Symptom duration ≥ six weeks | Joint  MCP 3 GS  MCP 5 PD  PIP 1 PD  PIP 3 GS  MTP 2 PD  MTP 5 PD  Wrist joint PD  Elbow GS  Tendon  Digit flexor tendon GS  Wrist ECU GS |

# Supplementary table 17 Principal component analysis of clinical and serological variables for seronegative patients

| **Variables** | **Component** | |
| --- | --- | --- |
|  | **1** | **2** |
| Symptom duration ≥ six weeks | .805 |  |
| Age ≥ 60 years old | .630 |  |
| Early morning stiffness of ≥ 60 mins |  | .869 |
| Tender joint count of 68 (0, 1-5 ≥ 6) | .495 | .585 |

Rotation Method: Varimax with Kaiser Normalization. Factor loadings of <0.400 are suppressed to facilitate interpretation. The variable with the highest loading factor from each component is highlighted in bold.

# Supplementary table S18 Principal component analysis of ultrasound variables for seronegative patients

| **Ultrasound variables** | **Component** | | | | | | |
| --- | --- | --- | --- | --- | --- | --- | --- |
|  | **1** | **2** | **3** | **4** | **5** | **6** | **7** |
| **MCP1 GS** | .824 |  |  |  |  |  |  |
| **MCP3 PD** | .813 |  |  |  |  |  |  |
| **MCP3 GS** | .802 |  |  |  |  |  |  |
| **MCP1 PD** | .799 |  |  |  |  |  |  |
| **MCP4 GS** | .784 |  |  |  |  |  |  |
| **MCP2 PD** | .781 |  |  |  |  |  |  |
| **MCP4 PD** | .754 |  |  |  |  |  |  |
| **MCP2 GS** | .748 |  |  |  |  |  |  |
| **MCP5 GS** | .518 |  |  |  |  |  |  |
| **PIP1 PD** |  | .905 |  |  |  |  |  |
| **PIP1 GS** |  | .905 |  |  |  |  |  |
| **MTP3 GS** |  | .610 |  |  |  |  |  |
| **MTP2 PD** |  | .538 |  |  | .462 |  |  |
| **PIP2 GS** |  |  | .914 |  |  |  |  |
| **PIP2 PD** |  |  | .907 |  |  |  |  |
| **PIP3 GS** |  |  | .701 |  |  |  |  |
| **Wrist flexor tendon GS** |  |  |  | .883 |  |  |  |
| **Wrist flexor tendon PD** |  |  |  | .870 |  |  |  |
| **Digit flexor tendon GS** |  |  |  |  | .829 |  |  |
| **Digit flexor tendon PD** |  |  |  |  | .824 |  |  |
| **Wrist GS** |  |  |  |  |  | .900 |  |
| **Wrist PD** |  |  |  |  |  | .900 |  |
| **Elbow GS** |  |  |  |  |  |  | .919 |
| **Elbow PD** |  |  |  |  |  |  | .874 |

Rotation Method: Varimax with Kaiser Normalization. Factor loadings of <0.400 are suppressed to facilitate interpretation. The variable with the highest loading factor from each component is highlighted in bold.

# Supplementary table 19 Summary of PCA variables for seronegative patients

| **PCA of clinical and serological variables** | | | | | | | |
| --- | --- | --- | --- | --- | --- | --- | --- |
| **Components** | **1** | | | **2** | | | |
| Variables | Symptom duration ≥ six weeks  Age ≥ 60 years old  Tender Joint Count of 68 | | | Early morning stiffness of ≥ 60 minutes  Tender joint count of 68 (0-1, 2-5 ≥ 6) | | | |
| % of variance explained | 35.802 | | | 27.392 | | | |
| Cumulative of variance explained | 63.194 | | | | | | |
| **PCA of ultrasound variables** | | | | | | | |
| **Components** | **1** | **2** | **3** | **4** | **5** | **6** | **7** |
| Variables | MCP1  MCP2  MCP3  MCP4  MCP5 | PIP1  MTP3  MTP2 | PIP2  PIP3 | Wrist flexor tendon | Digit flexor tendon  MTP2 | Wrist joint | Elbow |
| % of variance explained | 38.286 | 9.978 | 8.581 | 7.168 | 6.036 | 5.628 | 4.835 |
| Cumulative of variance explained | 80.511 | | | | | | |

# Supplementary table S20 Variables included in the forward stepwise logistic regression model for seronegative patients

| **Clinical and serological variables** | **US variables** |
| --- | --- |
| Symptom duration ≥ six weeks  Early morning stiffness of ≥ 60 minutes | Joint  MCP1GS  PIP1PD  PIP2GS  Wrist GS  Elbow GS  Tendon  Wrist flexor tendon GS  Digit flexor tendon GS |

# Supplementary table 21 Logistic regression model for seronegative patients

| **Variable** | **Odds ratio** | **95% CI** | **p-value** | **Nagelkerke R^2^** |
| --- | --- | --- | --- | --- |
| PIP 2 GS | 5.729 | 1.384 - 23.716 | .016 | 0.304 |
| Digit flexor tendon GS | 4.716 | 1.405 - 15.823 | .012 |  |
